# Identifying Digital Markers of Attention-Deficit/Hyperactivity Disorder (ADHD) in a Remote Monitoring Setting: Prospective Observational Study

**DOI:** 10.2196/54531

**Published:** 2025-01-29

**Authors:** Heet Sankesara, Hayley Denyer, Shaoxiong Sun, Qigang Deng, Yatharth Ranjan, Pauline Conde, Zulqarnain Rashid, Philip Asherson, Andrea Bilbow, Madeleine J Groom, Chris Hollis, Richard J B Dobson, Amos Folarin, Jonna Kuntsi

**Affiliations:** 1 Department of Biostatistics & Health Informatics, Institute of Psychiatry, Psychology and Neuroscience, King's College London, 16 De Crespigny Park, London, SE5 8AB, United Kingdom, 44 2078365454; 2 Social, Genetic and Developmental Psychiatry Centre, Institute of Psychiatry, Psychology and Neuroscience, King's College London, London, United Kingdom; 3 Department of Computer Science, University of Sheffield, Sheffield, United Kingdom; 4 Attention Deficit Disorder Information and Support Service (ADDISS), Edgware, Middlesex, United Kingdom; 5 School of Medicine, Mental Health & Clinical Neurosciences, Institute of Mental Health, University of Nottingham, Nottingham, United Kingdom; 6 NIHR MindTech Healthcare Technology Co-operative, Institute of Mental Health, University of Nottingham, Nottingham, United Kingdom; 7 Institute of Health Informatics, University College London, London, United Kingdom; 8 NIHR Biomedical Research Centre, South London and Maudsley NHS Foundation Trust and King’s College London, London, United Kingdom; 9 Health Data Research UK London, University College London, London, United Kingdom; 10 NIHR Biomedical Research Centre, University College London Hospitals NHS Foundation Trust, London, United Kingdom

**Keywords:** ADHD, smartphones, wearable devices, mobile health, mHealth, remote monitoring, surveillance, digital markers, attention-deficit/hyperactivity disorder, behavioral data, real world, adult, adolescent, participants, digital signals, restlessness, severity, predicting outcomes

## Abstract

**Background:**

The symptoms and associated characteristics of attention-deficit/hyperactivity disorder (ADHD) are typically assessed in person at a clinic or in a research lab. Mobile health offers a new approach to obtaining additional passively and continuously measured real-world behavioral data. Using our new ADHD remote technology (ART) system, based on the Remote Assessment of Disease and Relapses (RADAR)–base platform, we explore novel digital markers for their potential to identify behavioral patterns associated with ADHD. The RADAR-base Passive App and wearable device collect sensor data in the background, while the Active App involves participants completing clinical symptom questionnaires.

**Objective:**

The main aim of this study was to investigate whether adults and adolescents with ADHD differ from individuals without ADHD on 10 digital signals that we hypothesize capture lapses in attention, restlessness, or impulsive behaviors.

**Methods:**

We collected data over 10 weeks from 20 individuals with ADHD and 20 comparison participants without ADHD between the ages of 16 and 39 years. We focus on features derived from (1) Active App (mean and SD of questionnaire notification response latency and of the time interval between questionnaires), (2) Passive App (daily mean and SD of response time to social and communication app notifications, the SD in ambient light during phone use, total phone use time, and total number of new apps added), and (3) a wearable device (Fitbit) (daily steps taken while active on the phone). Linear mixed models and *t* tests were employed to assess the group differences for repeatedly measured and time-aggregated variables, respectively. Effect sizes (*d*) convey the magnitude of differences.

**Results:**

Group differences were significant for 5 of the 10 variables. The participants with ADHD were (1) slower (*P*=.047, *d*=1.05) and more variable (*P*=.01, *d*=0.84) in their speed of responding to the notifications to complete the questionnaires, (2) had a higher SD in the time interval between questionnaires (*P*=.04, *d*=1.13), (3) had higher daily mean response time to social and communication app notifications (*P*=.03, *d*=0.7), and (4) had a greater change in ambient (background) light when they were actively using the smartphone (*P*=.008, *d*=0.86). Moderate to high effect sizes with nonsignificant *P* values were additionally observed for the mean of time intervals between questionnaires (*P*=.06, *d*=0.82), daily SD in responding to social and communication app notifications (*P*=.05, *d*=0.64), and steps taken while active on the phone (*P*=.09, *d*=0.61). The groups did not differ in the total phone use time (*P*=.11, *d*=0.54) and the number of new apps downloaded (*P*=.24, *d*=0.18).

**Conclusions:**

In a novel exploration of digital markers of ADHD, we identified candidate digital signals of restlessness, inconsistent attention, and difficulties completing tasks. Larger future studies are needed to replicate these findings and to assess the potential of such objective digital signals for tracking ADHD severity or predicting outcomes.

## Introduction

The core clinical symptoms of attention-deficit/hyperactivity disorder (ADHD), which affects 5.9% of children and 2.5% of adults, are inattention, impulsivity, and hyperactivity [1]. The diagnosis relies on a semistructured interview designed to evaluate the diagnostic criteria for ADHD symptoms and associated functional impairments. Cognitive tasks, which are often used in research, also reveal behavioral responses that suggest lapses in attention or impulsive responses in people with ADHD [2-4]. For example, ADHD is strongly associated with increased reaction time variability: people with ADHD are highly inconsistent in their speed of responding to cognitive tasks, which is linked to difficulties with the regulation of attention and arousal [5,6].

Beyond the conventional in-person clinic- or lab-based assessments on clinical symptoms and cognitive functioning, the field of mobile health (mHealth) offers a new approach to obtaining additional, more detailed, and longer-term behavioral data in the real world. Passive monitoring using smartphone sensors enables ongoing, objective, and unobtrusive data collection while the participant continues with their everyday activities [7-10]. The real-world behaviors captured using such passive monitoring may, for example, provide digital signals of ADHD severity or predict outcomes. Emerging evidence from research on other disorders, such as depression and schizophrenia, indicates the potential for passive smartphone data to identify markers related to symptom severity or warning signs of relapse [11,12].

We recently developed a new remote measurement technology system for adults and adolescents with ADHD (older than 16 years)—the ADHD remote technology (ART) system—which uses the Remote Assessment of Disease and Relapses (RADAR)–base mHealth platform [13]. RADAR-base is an open-source platform to leverage data from wearable devices and mobile technologies. It provides scalable and customizable capabilities for remote real-time data collection from a wide range of sources and apps, providing a unified system for researchers to store, manage, and analyze the collected data. The RADAR-base Passive App collects background data from several sensors on modern smartphones, such as ambient noise, ambient light, phone use information (eg, which apps have been used and for how long), passive audio, GPS (relative) location, local Bluetooth device connectivity, battery life, gyroscope, steps, and acceleration from smartphone sensors. The Active App collects data requiring conscious effort (eg, questionnaires and other tasks), which are customizable according to study requirements. The platform sends notifications via the Active App to remind study participants to fill out the questionnaires and other prompts; related timestamps are recorded when the notification is delivered when participants start filling out the questionnaires and finish them.

Data previously collected using the Passive App in the RADAR-CNS (Remote Assessment of Disease and Relapse – Central Nervous System) long-term remote monitoring project [14] on adults with major depressive disorder revealed digital biomarkers that have the potential to predict depression severity [15]. For example, analyses focusing on a median follow-up period of 4 months showed that nearby Bluetooth device count—a proxy for social isolation—was associated with depression questionnaire scores [16]. The nearby Bluetooth device count can reflect the participants’ social connections and interactions with family, friends, co-workers, and strangers. Therefore, the data can also reflect participants’ time at home, mobility, social isolation, working status, and the number of other Bluetooth devices in the house and working environment. Further analyses of geolocation data over 24 months showed that an increase in depression symptom severity was preceded by an increase in time spent at home and a reduction in the average time spent spread across different locations. These findings from the RADAR-major depressive disorder study illustrate how data from the Passive App can be used to identify digital markers of the severity of psychiatric symptoms, supporting its application to other disorders, such as ADHD.

**Figure 1.**
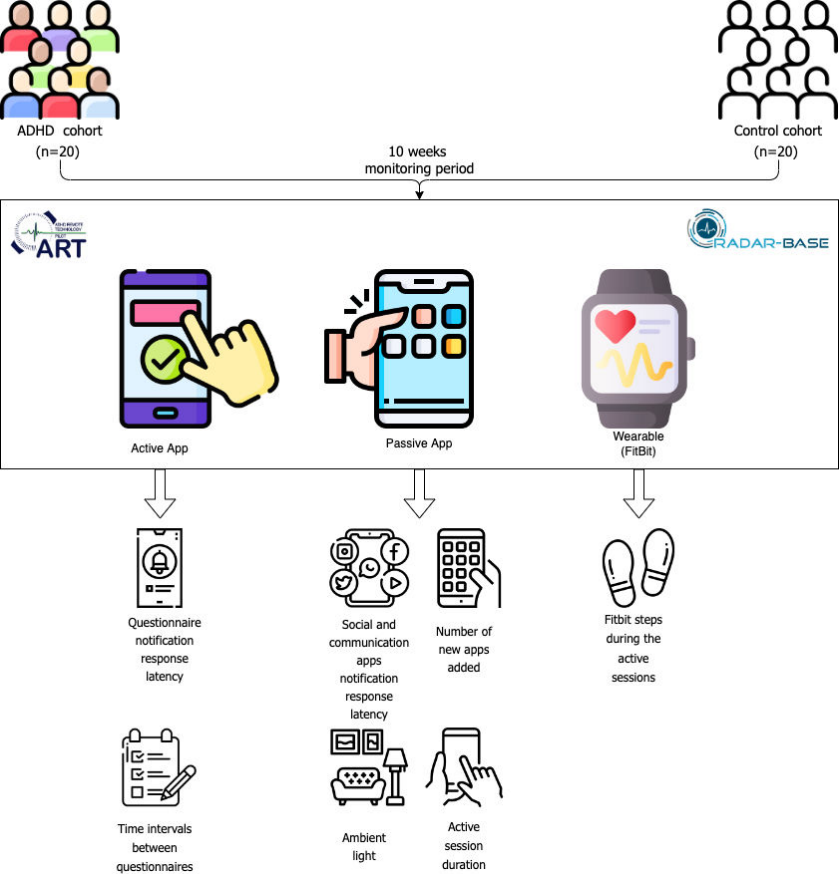
Graphical abstract showing study methodology and feature generation. A total of 20 participants with and 20 without ADHD were recruited in the United Kingdom between August and November 2020 and matched on age and gender. Participants completed the clinical symptom questionnaires via the Active App, while the smartphone sensor data were collected from the Passive App and the wearable sensor data from Fitbit. From the Active App data, the variables questionnaire notification response latency and time intervals between questionnaires are derived. Social and communication apps notification response latency, ambient light, number of new apps added, and active session duration are derived from the Passive App, whereas the Fitbit steps during the active sessions are derived from the wearable device. ART: attention-deficit/hyperactivity disorder remote technology; ADHD: attention-deficit/hyperactivity disorder; RADAR: Remote Assessment of Disease and Relapses.

We aimed to quantify and investigate whether adolescents and adults with ADHD differ from people without ADHD on a range of measures hypothesized to capture lapses in attention or restless or impulsive behaviors. As shown in Figure 1, we focus on analyzing passively collected data from the wearable device and a dedicated Passive App. Similar to the behavioral data gathered by the Passive App, the analysis of data from the Active App explores participants’ behavior during questionnaire completion. While the content of the questionnaires and cognitive data themselves are valuable for assessing ADHD and associated symptoms, they fall outside the scope of this particular paper. We aimed to investigate digital traces passively collected from participants’ smartphones and wearable devices during their daily lives.

## Methods

### Participants

Between August and November 2020, we recruited 20 individuals with ADHD and 20 comparison individuals without ADHD between the ages of 16 and 39 years into the study (Table 1). Participants were recruited from previous studies (where they had indicated that they were willing to be contacted regarding future research studies) via the Attention Deficit Disorder Information and Support Service using an advert placed on their website and emailed to members, social media (Facebook and Twitter), and placing an advert on the King’s College London Volunteer circular and on the “Call for Participants” website. Exclusion criteria were as follows: (1) having psychosis, major depression, mania, drug dependency, or a major neurological disorder; (2) any other major medical condition that might impact upon the individual’s ability to participate in normal daily activity; (3) pregnancy; and (4) IQ of <70. Participants in the ADHD group additionally met the diagnostic criteria for ADHD, and participants in the comparison group could not meet the diagnostic criteria for ADHD based on the self-report on the Barkley Adult ADHD Rating Scale (BAARS) on current symptoms (BAARS-IV) and Barkley ADHD functional impairment questionnaire.

**Table 1.** Demographics for the groups of participants with and without attention-deficit/hyperactivity disorder (ADHD).

Group	Female, n (%)	Age (years), mean (SD)	WASI-II^a^ vocabulary subscale, mean (SD)
ADHD (n=20)	15 (75)	27.49 (6.04)	57.85 (7.53)
Without ADHD (n=20)	15 (75)	27.79 (6.17)	56.80 (8.35)

a WASI-II: Wechsler Abbreviated Scale of Intelligence.

### Ethical Considerations

The study was approved by the North East – Tyne and Wear South Research Ethics Committee (REC reference: 20/NE/0034). Informed consent was obtained from participants before the assessments started. To maintain participant anonymity, their data were pseudonymized, and participants were identified in the study data with a code. Data collected from the apps, wearable devices, and interviews were only associated with this code and stored separately from any personally identifiable information. Participants were compensated £30 (US $37) after completion of the baseline sessions, £20 (US $25) after the first remote active monitoring follow-up (end of week 5), and a further £50 (US $62) at the study end point (end of week 10). The participants did not receive additional compensation for completing the debrief interviews.

### Procedure

ART is an observational, nonrandomized, noninterventional study using commercially available wearable technology and smartphone sensors, representing no change to the usual care or treatments of participants due to participation.

Participants attended 2 remote baseline sessions with a research worker using Microsoft Teams. The first remote baseline session with the participants with ADHD included the administration of the following assessments: (1) the Diagnostic Interview for ADHD in adults [17] to confirm ADHD diagnosis, (2) vocabulary and digit span subscales from the Wechsler Abbreviated Scale of Intelligence, and Wechsler Adult Intelligence Scale, respectively, and (3) web-based Research Electronic Data Capture (REDCap) [18] baseline questionnaires. The baseline REDCap questionnaires for the participants with ADHD included the About You (demographic) questionnaire, AQ-10 (autism), and the COVID-19 baseline questionnaire. The same was true for participants without ADHD however, in addition, they were required to complete the BAARS/Barkley Functional Impairment Scale (BFIS) (ADHD) to ensure they did not reach the research threshold for ADHD symptoms.

The second session was administered once participants had received their wearable device and smartphone by post, approximately a week after the first session. The second session included: (1) the administration of 2 cognitive tasks (combined cued continuous performance test and Go/NoGo task, and the Fast task) and (2) a training session on the use of the wearable device (Fitbit) and the smartphone Active and Passive Apps. The participant also received a leaflet summarizing key information (Participant Technology User Guide) and researcher contact details for future reference. Each session lasted for approximately 1.5 hours. Comparison participants were assessed in the same way, except that instead of the full ADHD diagnostic interview, they completed the ADHD symptom and impairment questionnaire [19].

During the baseline session, the participants with ADHD were asked to identify a partner, parent, or close friend who could complete informant-report versions of the active monitoring questionnaires on ADHD symptoms, impairment, and irritability using the web-based REDCap. The research worker contacted the informant and invited him/her to complete the questionnaires at each of the 3 remote active monitoring time points.

Each participant was, therefore, in the study for 10 weeks. The content of questionnaires and cognitive data are beyond the scope of this analysis. We describe below measures for variables used in this analyses.

### Measures

#### Active App: Clinical Questionnaires

Active monitoring involved the participant completing clinical symptom questionnaires on the RADAR-base smartphone Active App and 2 cognitive tasks on their home PC or laptop, which took place 3 times: at 2 weeks (the first remote self-administered assessment), 6 weeks (the second remote self-administered assessment) and 10 weeks (the third remote self-administered assessment) after the baseline remote researcher-led session.

Participants were requested to fill out the following questionnaires every 2 weeks: Patient Health Questionnaire depression scale[19], Affective Reactivity Index questionnaire [20], BAARS-IV [21], Generalized Anxiety Disorder-7 [22], and Reactive-Proactive Aggression Questionnaire [23]. Participants with ADHD also completed a medication use questionnaire; however, we excluded this questionnaire from our analyses, as the comparison group was not asked to complete it. Participants were informed that the questionnaires would be available for 3 days; however, they were told to complete them as soon as it was safe to do so. As the aim of this paper focuses on the passive measures of ADHD classification, the responses to the questionnaires are beyond the scope of this paper.

#### Passive Monitoring Measures

Passive monitoring, where data are collected without conscious effort from the participants using the smartphone and the wearable device (Fitbit Charge 3), started from the second baseline session and continued for 10 weeks for each participant. Passive data were collected continuously on a 24/7 basis. Participants with an existing Android phone had the option of either using their existing phone (1 participant with ADHD and 4 participants without ADHD) or upgrading it to a study Android phone (Motorola G7 Play or G7 Power; 3 participants with ADHD and 6 without ADHD). Participants who had an iPhone were asked to replace the iPhone with a study phone for the duration of the study (16 participants with ADHD and 10 without ADHD). Before enrolling in the study, participants were told they could only use an Android phone during the study period due to the availability of sensors and use data on Android devices. It was explained to them that due to our study design, they must only use one phone. During the study, the research team monitored phone battery status and phone app use to ensure compliance. Furthermore, we analyzed the battery discharging rate and found no significant differences in the discharging rate throughout the study period, confirming that the study phone was being used as the primary phone. Participants were asked to wear the wearable device on their nondominant hands.

The Passive App collected data on ambient noise, ambient light, phone use information (eg, which apps were used and how long, when the phone was unlocked), GPS location, Bluetooth connectivity, battery life, gyroscope, steps, and acceleration. Several passive measures were used to derive features to assess the smartphone use behavior of individuals with ADHD.

One such measure is phone status data. It logs the state of a smartphone at any given moment. The state can be “STANDBY” (device on standby), “UNLOCKED” (the device is unlocked), “SHUTDOWN” (the device is shut down), or “BOOTED” (the device is booting or starting). During the period between the device being unlocked and standby again, it is assumed that the participant is actively using the smartphone. We named this period an active phone use session.

The active phone use session can help us understand how long and when a participant is active on their smartphone. Another Passive App measure is the app event data. An “event” can represent any activity in the phone app. The app event data capture background (events that happen without the help of any active user interference), interactive (events that are initiated by the user using the app’s user interface), and foreground (events that are shown in the app notification section) events. The app event data can be used to understand the user’s smartphone activity (ie, what apps they use and how long they use them).

Combining this with the aforementioned active phone use data can be used to understand the smartphone behavior of individuals with ADHD. We calculated how long a participant took to open the phone after receiving a notification from any social and communication apps. We defined social and communication as applications that are labeled as “social” (Instagram, Facebook, etc) or “communication” (WhatsApp, Facebook Messenger, SMS, etc) in the Google Play store.

The smartphone also uses its light sensor to measure ambient light exposure level (ie, illuminance). We used the light sensors to assess the ambient information of participants. The data collection and management were handled by the open-source mHealth platform RADAR-base.

### Feature Generation

Using the measures described, we derived the following features that we hypothesize capture ADHD tendencies (Table 2).

**Table 2.** List of features for the analyses and their descriptions.

Number	Feature name	Feature description
1	Questionnaire notification response latency	The time taken by the participant to start completing the questionnaire after receiving the notification.
2	SD in questionnaire response latency	SD in the time taken by the participant to start completing the questionnaire after receiving the notification.
3	Mean interval between questionnaires	The mean time interval between finishing one questionnaire and starting the next one.
4	SD in intervals between questionnaires	SD in the time interval between finishing one questionnaire and starting the next one.
5	Daily mean of social and communication apps’ notification response latency	Daily mean of the response time to notifications from social and communication apps.
6	Daily SD in social and communication apps’ notification response latency	SD in the response time to notifications from the social and communication apps.
7	SD in ambient light	SD in ambient light while actively using the smartphone.
8	Fitbit steps during the active sessions	Fitbit step count during each active phone use session.
9	Active session duration	The time participant spends actively using their smartphone device in each active phone use session.
10	Number of new apps added	The total number of new apps added daily.

#### Questionnaire Response Time Features

The variable capturing the response latency of the questionnaire notification enables us to assess whether individuals with ADHD are slower to start answering the questionnaires following the notification. The variable mean intervals between questionnaires allow us to explore whether individuals with ADHD have a longer time interval between finishing one questionnaire and starting the next one. The SD in questionnaire response latency and intervals between questionnaires assess the variability in the response time and questionnaire intervals.

#### Passive Phone Use Features

We further created variables that allow us to assess whether participants with ADHD are more variable and differ overall in their speed of responding to notifications from social and communication apps. The notification data obtained using the Passive app is used to determine how individuals with ADHD respond to notifications from social and communication apps compared with the participants without ADHD.

When a notification from a social or communication app occurs, the phone will record an OTHER event type followed by a FOREGROUND or INTERACTION event if the participant has interacted with the notification. We have excluded all the notifications from the analyses that appeared when the participants were, indicated by their Fitbit data, to be sleeping to remove instances as it is highly unlikely that they could have seen the notifications. We analyzed the time it took for participants to unlock their phones after receiving a notification from a social or communication app.

Specifically, we computed the daily mean and SD of the notification data to derive the participants’ daily aggregate of smartphone behaviors.

#### Ambient Light Features

The variables on the SD in ambient light and steps while active on their smartphones were hypothesized to be related to restlessness by allowing us to assess whether individuals with ADHD move around more than participants without ADHD when they are actively using their devices.

#### Phone Activity Features

We also created variables measuring the length of each active phone use session and the number of new apps downloaded to assess whether shorter active phone use sessions and a greater number of new apps downloaded are observed in the participants with ADHD.

### Statistical Methods

We used linear mixed models (LMMs) to examine differences between the groups for repeatedly measured variables over the study period. These variables include questionnaire notification response latency, mean intervals between questionnaires, the SD in intervals between questionnaires, daily mean of social and communication apps’ notification response latency, the daily SD in social and communication apps’ notification response, the SD in ambient light, Fitbit steps during the active sessions, active session duration and the number of new apps added.

We chose this approach as it takes the nonindependence of data into account. Therefore, we can use repeated measures from the same participant. LMMs are also highly robust and do not need to satisfy the prerequirements necessary for ANOVA models [24]. Moreover, we investigated the interaction effect between the days since the recruitment and the control group with respect to questionnaire notification response latency and intervals between questionnaires to evaluate how being in the study for a longer duration impacts these behavioral variables.

For nontemporal aggregated variables (SD in questionnaire notification response latency), we used the standard *t* test to compare the group differences. The *P* values are noted by *P* in the text, and variables are defined as statistically significant if *P*<.05. Along with *P* values, we have also reported the Standard Score (Z value) and effect size (*d*). For repeatedly measured variables where we used LMM, we have computed the effect size in Formula 1 [25]. The effect size of nontemporal aggregated variables is computed using Cohen *d* [26].

Formula 1, measuring effect sizes on the results of LMM [25], is as follows:


$$d=\frac{difference\;between\;means}{\sqrt{varintercept_{part}+varintercept_{item}+varslope_{part}+varslope_{item}+var_{residual}}}$$

where

*d*=effect size

$varintercept_{part}$=the intercept coefficient per participant

$varintercept_{item}$=the intercept coefficient per item

$varslope_{part}$=the slope coefficient per participant

$varslope_{item}$=the slope coefficient per item

$var_{residual}$=the residual variance coefficient

The effect size *d*>0.6 has been employed to determine nonsignificant trends [27]. The cut-off was used to correspond to moderate to high effect sizes. As this is primarily an exploratory study and to inform future larger-scale studies, multiple testing corrections were not undertaken to reduce the chance of type-two errors (ie, false negative results) and to avoid over-correction for multiple comparisons involving multiple correlated variables.

We identified and removed outliers by using the IQR proximity rule, a well-established method for outlier detection in skewed distributions [28]. We calculated the upper limit (UL) and the lower limit (LL), a statistical threshold computed from data points according to Formula 2. Any values in the data above UL and below LL are deemed outliers and removed from the analyses. The derived limits serve as a reference point to identify and manage outliers or unusual data points in a data set. To assess the influence of potential outliers, we conducted a sensitivity analysis. This involved reanalyzing the data after excluding the 2 participants with the highest SD in ambient light values.

Formula 2, the IQR proximity rule [28], is as follows:


data:List of data points

Q1=Percentile(data,25)

Q3=Percentile(data,75)

IQR=Q3−Q1

LL=Q1−1.5×ILR;UL=Q3+1.5×ILR

## Results

Group differences were significant for 5 out of the 10 variables: questionnaire notification response latency, the SD in questionnaire notification response latency, the SD in intervals between questionnaires, daily mean of social and communication apps’ notification response latency, and SD in ambient light (Table 3). While not significant, the mean intervals between questionnaires, the daily SD in social and communication apps’ notification response latency and Fitbit steps during the active session show results from moderate to high effect size, that is, *d*>0.60.

**Table 3.** Summary of results for all the 10 derived features.

Number	Feature name	ADHD^a^, mean (SD)	Without ADHD, mean (SD)	*P* value	Standard score (Z)	Effect size (*d*)
1	Questionnaire notification response latency (in hours)^b^	19.14 (12.98)	11.69 (10.31)	.047	−1.99	1.05
2	SD in questionnaire notification response latency (in hours)^b^	15.88 (9.27)	8.97 (6.83)	.01	2.68	0.84
3	Mean intervals between questionnaires (in hours)	1.12 (2.17)	0.18 (0.64)	.06	−1.18	0.82
4	SD in intervals between questionnaires (in hours)^b^	2.01 (5.04)	0.35 (2.23)	.04	−2.03	1.13
5	Daily mean of social and communication apps’ notification response latency (in seconds)^b^	2304.27 (1406.62)	1516.45 (785.78)	.03	−2.17	0.7
6	Daily SD in social and communication apps’ notification response latency (in seconds)	2736.38 (1091.17)	2125.15 (840.86)	.05	−1.96	0.64
7	SD in ambient light (in lux)^b^	83.96 (155.57)	43.63 (73.57)	.008	−2.67	0.86
8	Fitbit steps during the active sessions (in steps)	313.14 (263.88)	179.20 (177.26)	.09	−1.67	−0.61
9	Active session duration (in seconds)	1778.07 (1653.87)	1047.68 (1060.45)	.11	−1.60	0.54
10	Number of new apps added	1.18 (0.40)	1.43 (0.81)	.24	1.18	0.18

a ADHD: attention-deficit/hyperactivity disorder.

b Statistically significant result.

For questionnaire notification response latency, mean intervals between questionnaires and SD in intervals between questionnaires, we observed the interaction of these features with time and found that the features increased slightly, though non significantly, with each day. We did not find any significant interaction between the control group and the day for the features. The negative coefficient observed corresponding to the interaction effect for the features implies that the increase in features per day is smaller in the comparison group compared with the ADHD group.

**Figure 2.**
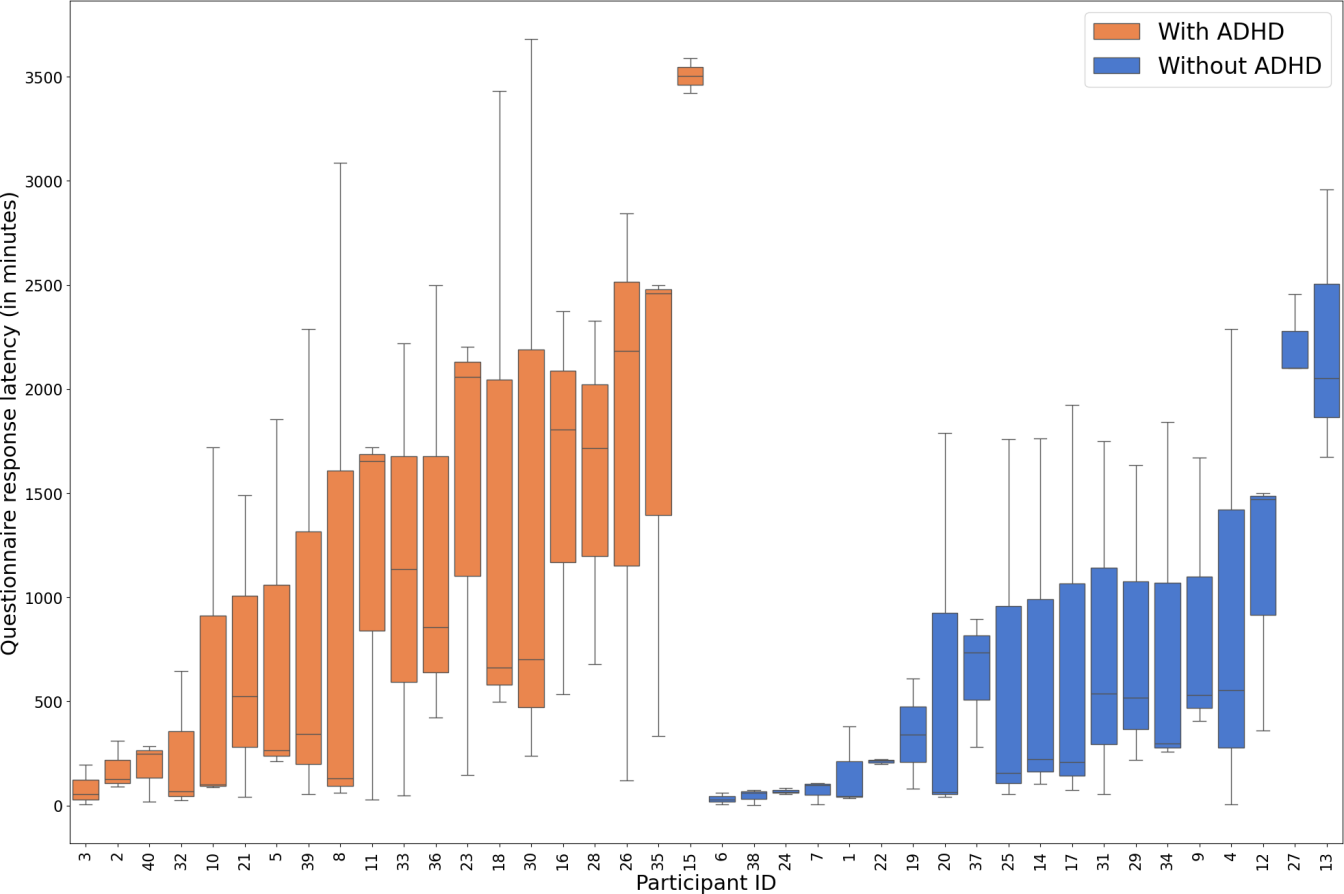
The figure presents the differences in the time taken by the participant to start completing the questionnaire after receiving the notification (questionnaire notification response latency) in participants with and without ADHD. ADHD: attention-deficit/hyperactivity disorder.

**Figure 3.**
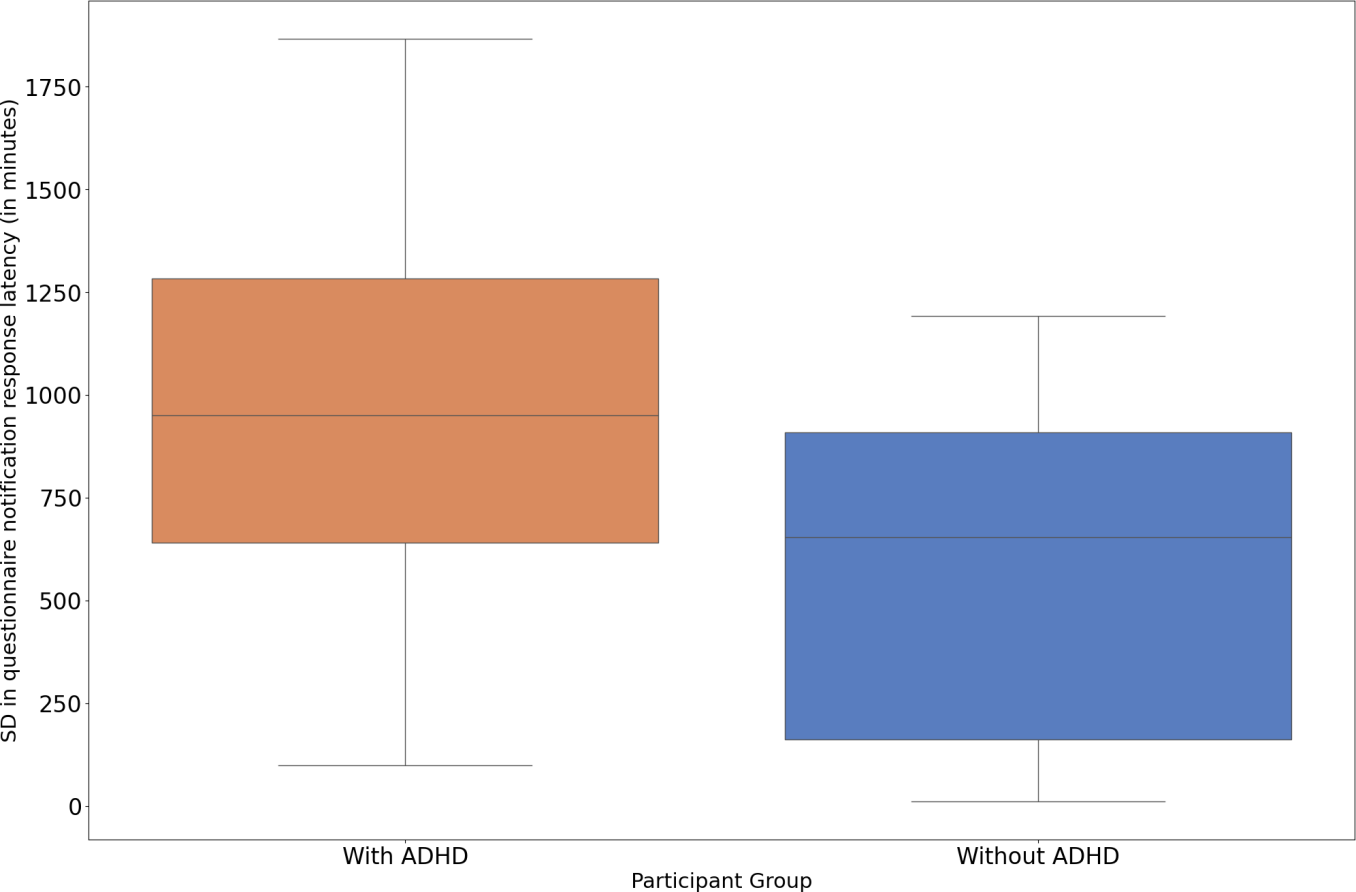
SD in the time taken by the participant to start completing the questionnaire after receiving the notification (SD in questionnaire notification response latency) in individuals with and without ADHD. The boxplot represents the median and IQR of SD in questionnaire notification response latency for both groups. ADHD: attention-deficit/hyperactivity disorder.

**Figure 4.**
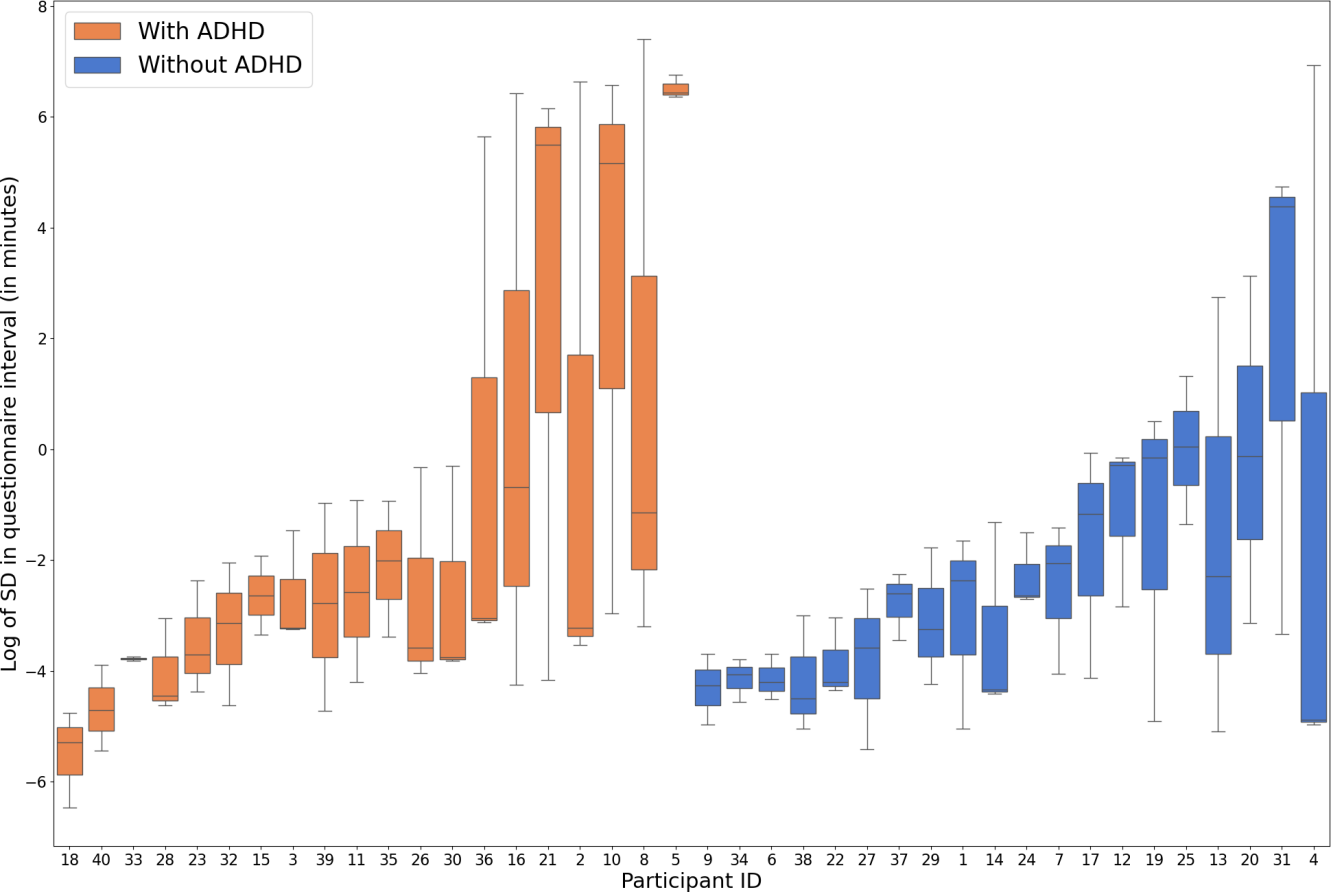
Log of SD in the interval between finishing one questionnaire and starting the next one (SD in intervals between questionnaires) in participants with and without ADHD. ADHD: attention-deficit/hyperactivity disorder.

**Figure 5.**
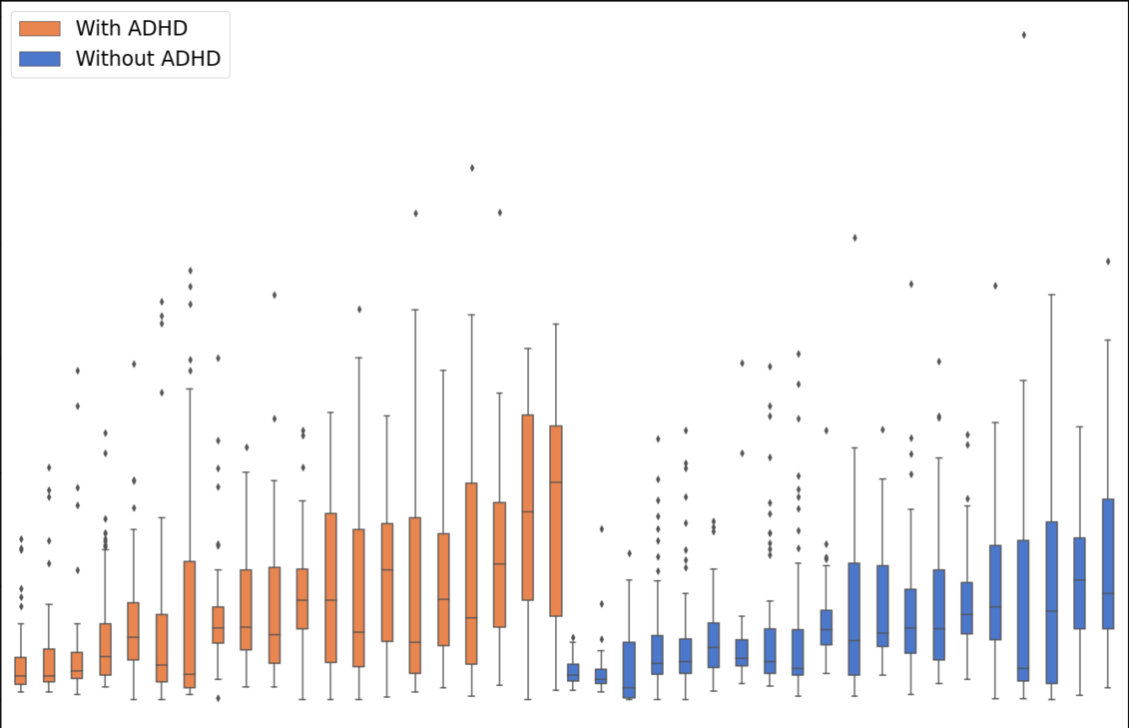
Daily mean of response time to notifications from social and communication apps (daily mean of social and communication apps’ notification response latency) in participants with and without ADHD. ADHD: attention-deficit/hyperactivity disorder.

**Figure 6.**
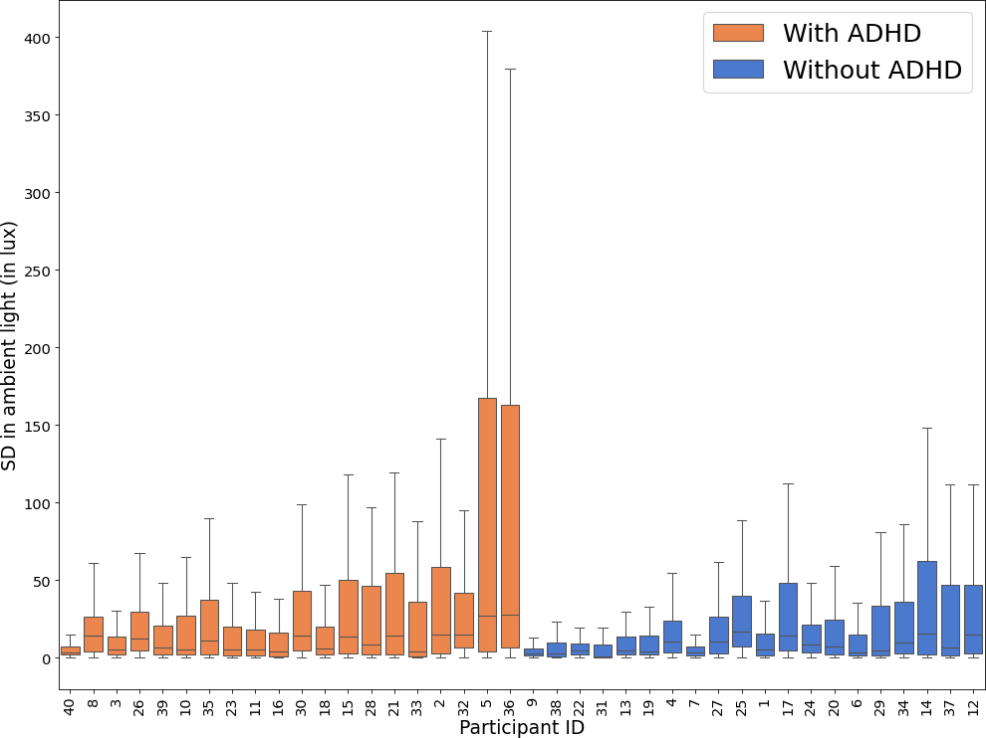
SD in ambient light while actively using the smartphone in participants with and without ADHD. ADHD: attention-deficit/hyperactivity disorder.

Figures 2-6 illustrate the results for questionnaire notification response latency, SD in questionnaire notification response latency, SD in intervals between questionnaires, daily mean of social and communication apps’ notification response latency, and SD in ambient light, respectively. Figure 5 shows the SD in ambient light while participants are actively using their smartphones. This reveals that participant IDs 5 and 36, who have ADHD, are potential outliers. The sensitivity analysis confirmed that the group difference remained statistically significant after these exclusions. Therefore, we have chosen to include all participants in the final analysis for transparency and robustness.

## Discussion

### Principal Findings

In a novel exploration of digital markers of ADHD, we identified candidate digital signals of restlessness, inconsistent attentional focusing, and difficulties completing tasks as part of a 10-week remote monitoring pilot study on our ART system.

Analyses of the data from the smartphone Active App, where participants were requested to complete a series of questionnaires at 4-week intervals, revealed that individuals with ADHD were slower and more variable in starting to complete questionnaires after receiving a notification, and more variable in the time interval between finishing one questionnaire and starting the next one, than the participants without ADHD. The Passive App data further showed that the participants with ADHD were slower to open their smartphones following a notification from a social or communication app compared with the group without ADHD. These novel findings—as well as further potential signals that emerged at trend-level only (greater mean in the ADHD group in the intervals between questionnaires and variability in the time taken to open the phone after receiving a social or communication app notification)—await replication in larger future studies.

The above findings are in line with the frequently reported difficulties in completing tasks among people with ADHD [3] and may further reflect characteristic inconsistent attentional focusing. Future research is needed to explore whether the slower and more variable speed of responding captured on the smartphone data reflects the same difficulties with the regulation of attention and arousal reported in lab-based cognitive studies in individuals with ADHD. Such cognitive studies strongly link ADHD with inconsistency in the speed of responding to cognitive tasks, which has been linked in neurophysiological studies of the regulation of attention and arousal [5,6]. Whether passive remote data collection using smartphones could offer a real-world passive alternative or augmentation to the more intensive cognitive task administration to measure behavioral characteristics associated with ADHD requires careful further study. Given the general availability of smartphones in the general population, the possibility of using this approach for symptom monitoring remains intriguing.

An additional group difference from our analyses of the Passive App data was the SD in ambient (background) light concurrent with the use of their mobile phone, which was significantly higher among the participants with ADHD. Further investigation would be useful to understand this better, but we presently hypothesize it may relate to participants with ADHD moving between differentially lit environments while using their smartphones. A related group comparison on Fitbit step count while active on their smartphone was not significant, but the high effect size observed again emphasizes the need for future research with increased statistical power. We did not observe a difference between participants with and without ADHD in the number of new apps downloaded or in the length of active smartphone use sessions, which suggests that these markers do not capture impulsivity, restlessness, or other symptoms of ADHD.

### Limitations

Since this was a pilot study, the sample size was small (n=40), and the findings require replication in future studies with larger samples. While this sample size provided sufficient power to explore initial relationships between ADHD and phone-based behavioral markers, it limits the generalizability of the findings to the broader ADHD population. Smaller sample sizes can increase the likelihood of chance findings and decrease the ability to detect subtle but meaningful effects. Therefore, replicating these findings in larger and more diverse samples is necessary to confirm their validity and generalizability. A strength of our design was, however, the large amount of data collected per participant: 10 weeks’ worth of multimeasure remote monitoring data, which, in the case of passive monitoring data, was collected 24 hours a day. During the 10 weeks of the study period, we collected an average of 99,180 Fitbit step data points per participant. We collected an average of 149,821 different app activities per participant from 1855 unique apps during the same period.

Another limitation is that the data were collected during the COVID pandemic when restrictions were in place that may have affected daily activities (eg, physical activity, sleep, and smartphone activity) and well-being more generally. Importantly for our analyses, however, we note that such restrictions were concurrent for both groups.

Another limitation to consider is that 75% of our participants are women, and hence, there is a gender imbalance in our study. However, the groups were matched on gender. Future larger studies should aim to recruit samples with more representative gender distributions.

Furthermore, we provided several participants (n=35) who owned an Apple smartphone or a noncompatible Android smartphone with a new compatible Android smartphone. Despite instructing participants to use the new Android phone as their primary phone during their remote monitoring period, as participants had access to their Apple smartphone and noncompatible smartphone, it is difficult to assess how much they may have used their nonstudy phones.

### Future Research

We are currently conducting a large remote monitoring study on adults with ADHD—the ADHD remote technology study of cardiometabolic risk factors and medication adherence (ART-CARMA) [29]. In ART-CARMA, we obtain remote monitoring data from the participants with ADHD both before and after initiation of ADHD medication treatment, which will allow us to examine the effects of medication on the digital markers of ADHD that we identified here. In total, we will obtain remote monitoring data from each participant for 12 months, enabling detailed over-time analyses of the multiple measures. Another direction for future research would be to explore potential age- and gender-related differences in digital markers, from childhood through adolescence and young adulthood, to older adulthood, in both men and women.

### Conclusions

Using the ART system in a 10-week remote monitoring study of participants with and without ADHD, we identified novel candidate digital markers of ADHD: digital signals that we propose to capture ADHD symptoms of restlessness, inconsistent attentional focusing, and difficulties completing tasks. Future research can assess the potential for the candidate digital markers of ADHD, as identified here, to track ADHD severity or predict outcomes.

## Abbreviations

ADHD: attention-deficit/hyperactivity disorder
ART: attention-deficit/hyperactivity disorder remote technology
ART-CARMA: attention-deficit/hyperactivity disorder remote technology study of cardiometabolic risk factors and medication adherence
BAARS: Barkley Adult Attention-Deficit/Hyperactivity Disorder Rating Scale
BFIS: Barkley Functional Impairment Scale
LL: lower limit
LMM: linear mixed model
mHealth: mobile health
RADAR: Remote Assessment of Disease and Relapses
REDCap: Research Electronic Data Capture
UL: upper limit
